# Essential role of the Dishevelled DEP domain in a Wnt-dependent human-cell-based complementation assay

**DOI:** 10.1242/jcs.195685

**Published:** 2016-10-15

**Authors:** Melissa V. Gammons, Trevor J. Rutherford, Zachary Steinhart, Stephane Angers, Mariann Bienz

**Affiliations:** 1 MRC Laboratory of Molecular Biology, Cambridge Biomedical Campus, Francis Crick Avenue, Cambridge CB2 0QH, UK; 2 Leslie Dan Faculty of Pharmacy, Room 901, University of Toronto, 144 College Street, Toronto, Ontario, CanadaM5S 3M2

**Keywords:** Dishevelled, DEP domain, PDZ domain, Frizzled

## Abstract

Dishevelled (DVL) assembles Wnt signalosomes through dynamic head-to-tail polymerisation by means of its DIX domain. It thus transduces Wnt signals to cytoplasmic effectors including β-catenin, to control cell fates during normal development, tissue homeostasis and also in cancer. To date, most functional studies of Dishevelled relied on its Wnt-independent signalling activity resulting from overexpression, which is sufficient to trigger polymerisation, bypassing the requirement for Wnt signals. Here, we generate a human cell line devoid of endogenous Dishevelled (DVL1– DVL3), which lacks Wnt signal transduction to β-catenin. However, Wnt responses can be restored by DVL2 stably re-expressed at near-endogenous levels. Using this assay to test mutant DVL2, we show that its DEP domain is essential, whereas its PDZ domain is dispensable, for signalling to β-catenin. Our results imply two mutually exclusive functions of the DEP domain in Wnt signal transduction – binding to Frizzled to recruit Dishevelled to the receptor complex, and dimerising to cross-link DIX domain polymers for signalosome assembly. Our assay avoids the caveats associated with overexpressing Dishevelled, and provides a powerful tool for rigorous functional tests of this pivotal human signalling protein.

## Abstract (precis)

**Summary:** A physiological complementation assay in Dishevelled null-mutant human cells establishes an essential function of the DEP domain of Dishevelled in its binding to Frizzled for signal transduction to β-catenin.

## INTRODUCTION

The Wnt/β-catenin cascade is an ancient signalling pathway that specifies numerous cell fates during embryonic development and tissue homeostasis of animals, and its aberrant activation leads to various cancers, most notably colorectal cancer (Clevers and Nusse, 2012). Its key effector, β-catenin, is continuously destabilised by the Axin degradasome, which also contains the APC tumour suppressor and two serine-threonine protein kinases (glycogen synthases kinase 3, GSK3; casein kinase 1α, CK1α) that phosphorylate the N-terminus of β-catenin to earmark it for proteasomal degradation (MacDonald et al., 2009). This process is inhibited when a Wnt protein couples a cognate Frizzled receptor (FZD1–FZD10 in humans; Fz1–Fz4 in flies) with its low-density lipoprotein receptor-related protein 5/6 co-receptor (typically LRP6) (Cong et al., 2004; Tamai et al., 2000), which triggers recruitment of Dishevelled (DVL) to the plasma membrane. DVL thus assembles a signalosome that engages the Axin degradasome to block the enzymatic activity of GSK3 within this complex (Metcalfe and Bienz, 2011; Piao et al., 2008; Wu et al., 2009). This allows β-catenin to accumulate and to bind to TCF/LEF factors in the nucleus, to operate context-dependent transcriptional switches that determine specific cell fates during normal development and in neoplasia (Mosimann et al., 2009).

DVL is an ancient signalling hub protein found in all animals and in humans. It functions as a pivot in transducing Wnt signals either to β-catenin to effect nuclear Wnt responses, or to various alternative cytoplasmic effectors that elicit ‘non-canonical’ Wnt responses, typically in post-mitotic cells, specifying cellular features such as planar cell polarity (PCP) (Angers and Moon, 2009; Wynshaw-Boris, 2012). Animals encode between one and three DVL paralogs (DVL1–DVL3 in humans), typically with redundant functions. Each paralog has three structured domains – DIX (Dishevelled and Axin) at its N-terminus, PDZ (Post-synaptic density protein-95, Disc large tumour suppressor, Zonula occludens-1) and DEP (Dishevelled, Egl-10 and Pleckstrin) – separated from each other by long flexible linkers. Various functions and ligands have been ascribed to these domains, typically based on overexpression assays.

The DIX domain has a unique activity in undergoing dynamic and reversible head-to-tail polymerisation resulting in meta-stable filaments, which enables Dishevelled to assemble signalosomes (Schwarz-Romond et al., 2007a). These are dynamic protein assemblies that are detectable in cells by immunofluorescence as distinct membrane-associated or cytoplasmic puncta (Schwarz-Romond et al., 2005). DIX-dependent polymerisation causes a dramatic increase in the local concentration of Dishevelled, which increases its avidity for low-affinity binding partners, thus allowing Dishevelled to interact efficiently with signalling effectors (Bienz, 2014). One of these is Axin whose own DIX domain mediates its co-polymerisation with Dishevelled through direct heterotypic DIX–DIX interactions (Fiedler et al., 2011). Polymerised Dishevelled also promotes the phosphorylation of the cytoplasmic tail of LRP6 (Bilic et al., 2007), to generate phosphorylated PPPSPxS/T motifs, which serve as high-affinity docking sites for Axin (Tamai et al., 2004) and inhibit GSK3 by binding to its catalytic pocket (Stamos et al., 2014), thereby stabilising β-catenin and transducing Wnt signals to the nucleus. Dishevelled signalosomes are also likely to transduce non-canonical Wnt signals, given that these responses seem to be contingent on membrane-associated Dishevelled puncta (e.g. Axelrod, 2001; Park et al., 2005). We have recently discovered that the signalling activity of Dishevelled also depends on its DEP domain, which dimerises, probably to crosslink DIX polymers to catalyse signalosome assembly (Gammons et al., 2016).

The PDZ and DEP domains have both been implicated in the binding to Frizzled receptors. Frizzled proteins are distantly related to the G-protein coupled receptor (GPCR) family (forming their fourth class, class F; Venkatakrishnan et al., 2013), and their cytoplasmic tail contains a highly conserved KTxxxW motif that is essential for Wnt signal transduction to β-catenin (Umbhauer et al., 2000), and also for non-canonical Wnt responses (Wu et al., 2008). Evidence from biophysical *in vitro* binding assays based on recombinant protein suggested that this motif can interact directly with the PDZ (Wong et al., 2003) or the DEP domain (Tauriello et al., 2012). Cell-based assays provided evidence for a functional interaction between Frizzled and DEP but not PDZ (Pan et al., 2004; Tauriello et al., 2012; Gammons et al., 2016). However, definitive proof for the function of these domains in Frizzled binding is still missing as the signalling by Dishevelled in these overexpression assays does not depend on physiological Wnt signals.

The reason for this is that overexpression of Dishevelled alone is sufficient to trigger its polymerisation (Schwarz-Romond et al., 2007a), which initiates signal transduction to β-catenin through Axin co-polymerisation (Fiedler et al., 2011; Schwarz-Romond et al., 2007b). Thus, these activities of overexpressed Dishevelled neither require Wnt nor its recruitment to Frizzled, although they depend at least partially on the LRP6 co-receptor, both in mammalian cells and in flies (Metcalfe et al., 2010). Indeed, it is well-established that Dishevelled overexpression not only activates signalling through β-catenin, but also interferes with PCP and other non-canonical signalling responses both in vertebrate and invertebrate tissues (e.g. Boutros et al., 1998; Park et al., 2005; Penton et al., 2002). This imposes a caveat on the conclusions that can be derived from these overexpression studies, in particular those regarding the functional interaction of Dishevelled with Frizzled upon Wnt signalling – the crucial step initiating Wnt signal transduction.

Here, we have developed a complementation assay based on human embryonic kidney (HEK293T) cells whose three DVL paralogs were deleted by CRISPR–Cas9 technology (DVL triple-knockout, TKO). These DVL TKO cells are unable to stabilise β-catenin in response to Wnt and to activate β-catenin-dependent transcription, but these responses can be restored by re-expression of DVL2 at near-endogenous levels. Using this assay to test various mutants of DVL2, we demonstrate that its DEP-dependent binding to Frizzled is essential for Wnt-dependent signalling to β-catenin, whereas its PDZ domain is dispensable for β-catenin responses. This establishes two mutually exclusive functions of the DEP domain, switching between binding to Frizzled and cross-linking Dishevelled polymers for signalosome assembly. Our complementation assay opens up further studies of the function of this pivotal signalling protein, and might also be potentially adaptable for the study of non-canonical Wnt responses.

## RESULTS

### DVL TKO cells fail to respond to Wnt

**Fig. 1.**
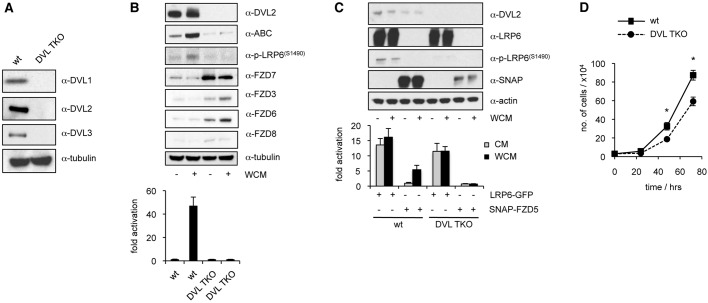
**Dishevelled TKO cells fail to respond to Wnt3a.** (A) Western blot analysis of lysates from DVL TKO or parental control cells (wt), probed with antibodies as indicated. (B) Graph shows SuperTOP assays in DVL TKO or control cells, with or without 6 h of incubation in Wnt3a-conditioned medium (WCM). Above, corresponding western blots (with or without 2 h incubation in WCM). ABC, activated β-catenin. (C) Graph shows SuperTOP assays in DVL TKO or control cells (as in B), transiently transfected with LRP6–GFP or SNAP–FZD5 (lysed 24 h after transfection). Above, corresponding western blot. (D) Proliferation of DVL TKO or control cells. *n*=3 independent experiments, statistical test two-way ANOVA with repeated measures, *P<0.05. Error bars represent s.e.m. of three independent experiments.

We generated DVL TKO HEK293T cells using CRISPR–Cas9 technology, selecting individual cell clones that fail to express any of the three DVL paralog proteins, as judged by western blotting (Fig. 1A), and confirmed by sequencing of target sites. As expected, Wnt3a-treated DVL TKO cells (unlike their parental controls) failed to accumulate non-phosphorylated (‘active’) β-catenin (ABC) and to stimulate β-catenin-dependent transcription, as judged by SuperTOP luciferase reporter assays (Fig. 1B), demonstrating that DVL function is essential for these β-catenin responses. We also confirmed that these TKO cells exhibit increased levels of several FZD paralogs expressed by them (Fig. 1B), corroborating recent findings that Dishevelled promotes the destabilisation of Frizzled by RNF43 and/or ZNRF3, a pair of paralogous membrane-spanning E3 ubiquitin ligases that clear Frizzled from the plasma membrane in the absence of Wnt signalling (Jiang et al., 2015). Furthermore, phosphorylation of LRP6 S1490 (within the membrane-proximal PPPSP motif) is reduced to background levels in the TKO cells (Fig. 1B), confirming the key role of Dishevelled in conferring this Wnt-dependent phosphorylation of LRP6 (Bilic et al., 2007; Zeng et al., 2008).

As Dishevelled signals through LRP6 (Metcalfe et al., 2010), we wondered whether LRP6 overexpression might restore β-catenin responses in DVL TKO cells. This was the case; overexpression of LRP6–GFP, but not of SNAP–FZD5, stimulated β-catenin-dependent transcription in DVL TKO cells, regardless of Wnt3a (Fig. 1C), consistent with previous epistasis experiments (Cong et al., 2004). Note that there was very little S1490 phosphorylation in these experiments despite the high levels of LRP6 overexpression, corroborating the important role of Dishevelled in promoting this phosphorylation. However, the low level of Dishevelled-independent S1490 phosphorylation (probably resulting from clustering of the overexpressed LRP6; Metcalfe and Bienz, 2011) seemed to be sufficient for signalling to β-catenin, possibly because this phosphorylation within PPPSP primes for CK1-mediated phosphorylation of the +2 serine or threonine residue flanking these motifs (Davidson et al., 2005; Zeng et al., 2005), which might suffice for blocking GSK3. Conversely, overexpressed DVL2–GFP (giving rise to cytoplasmic puncta) is severely compromised in its ability to signal in LRP6 KO cells, regardless of Wnt stimulation (Fig. S1). These results corroborate the epistatic relationship between Dishevelled and LRP6 in human cells, establishing LRP6 as a key effector of Dishevelled during signal transduction to β-catenin.

Finally, we also noticed that DVL TKO cells proliferate more slowly than their parental control (Fig. 1D), consistent with the previously reported roles of Wnt signalling and Dishevelled in promoting cell cycle progression (Kikuchi et al., 2010; Niehrs and Acebron, 2012).

### Low levels of re-expressed DVL2 restores Wnt-dependent β-catenin responses

**Fig. 2.**
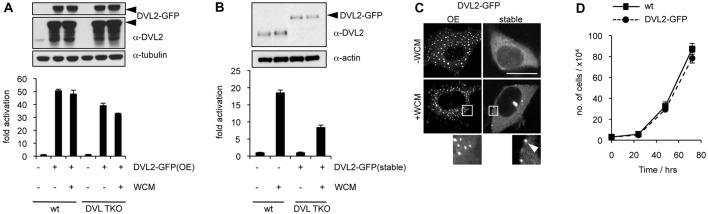
**Re-expressed DVL2 restores Wnt responses in DVL TKO cells.** (A,B) Graph shows SuperTOP assays in DVL TKO or control cells (A) transiently or (B) stably transfected with DVL2–GFP, *n*=3 independent experiments (as in Fig. 1C). Above, corresponding western blots. (C) Confocal images of representative DVL TKO cells transiently or stably expressing DVL2–GFP (fixed 18 h after transfection), as indicated (OE, overexpression). Lower panels show examples of cytoplasmic and plasma membrane-associated puncta (arrowhead) at higher magnification. Scale bar, 10 µm. (D) Proliferation assay of DVL TKO cells, with and without stable expression of DVL2–GFP. *n*=3 independent experiments, two-way ANOVA with repeated measures, *P<0.05. Error bars represent s.e.m. of three independent experiments.

We chose DVL2 to restore Wnt/β-catenin responses in DVL TKO cells as this is the predominant DVL paralog in many mammalian cells (e.g. Lee et al., 2008). Dvl2 is also crucial in murine development although its function is redundant with Dvl3 in several tissues (Etheridge et al., 2008; Hamblet et al., 2002), whereas Dvl1 is dispensable for normal development (Lijam et al., 1997). Re-supplying DVL2–GFP by transient transfection of DVL TKO resulted in high levels of β-catenin-dependent transcription, which was not further increased by Wnt stimulation (Fig. 2A), indicating efficient Wnt-independent signalling activity of this transgene. This is accompanied by cytoplasmic DVL2–GFP puncta that are visible virtually in every transfected cell (see below).

By contrast, β-catenin-dependent transcription is clearly Wnt3a-dependent if DVL TKO cells are supplied with low levels of DVL2–GFP by means of the pBABEpuro vector, which produces ∼10× induction of the β-catenin-dependent signalling activity, corresponding to ∼50% of the transcriptional response mounted by the parental HEK293T cells (Fig. 2B). This implies that the re-supplied DVL2–GFP signals as efficiently as endogenous DVL: the level of DVL2–GFP in the DVL TKO cells is roughly the same as that of the endogenous DVL2 in the parental cells (Fig. 2B), which, in addition, also express DVL1 and DVL3 (Fig. 1A). Importantly, DVL2–GFP is completely diffuse in the absence of Wnt3a, and the only puncta that can be detected occasionally in these cells are associated with the nuclear envelope, as previously observed in HEK293T cells expressing an inducible DVL2–GFP (Smalley et al., 2005), probably reflecting DVL2–GFP depositions at microtubule-organising centres. However, short pulses of Wnt3a produce a small number of membrane-associated puncta in a fraction of the cells (Fig. 2C, lower panels), reminiscent of the Wnt-dependent membrane-associated DVL puncta observed in zebrafish embryos (Hagemann et al., 2014).

The rate of proliferation of the DVL TKO line stably expressing DVL2–GFP is indistinguishable from that of its parental wild-type (wt) control (Fig. 2D), indicating that the DVL2–GFP rescue transgene restores normal proliferation in the DVL TKO cells. Notably, live-cell imaging of transiently transfected COS-7 cells revealed that DVL2–GFP continues to accumulate to high levels in these cells after transient transfection until they inevitably arrest, without ever progressing through mitosis (Movie 1). It is thus likely that the growth of the stably transfected DVL TKO cells depends on their ability to limit the expression of the DVL2–GFP rescue transgene. There seems to be negative selective pressure to ensure that the cellular concentration of DVL2–GFP is kept to near-physiological levels (see also below).

### The PDZ domain is dispensable for Wnt signal transduction to β-catenin

An early biochemical study provided evidence that a peptide spanning the KTxxxW motif from *Xenopus* Fz7 interacted with the purified PDZ domain from Dvl1 (Wong et al., 2003), implicating this domain in the recruitment of Dishevelled to the receptor complex in the plasma membrane. However, overexpressed PDZ fails to be recruited to FZD in cell-based assays (Pan et al., 2004; Tauriello et al., 2012; Gammons et al., 2016), questioning the physiological role of this *in vitro* interaction. To determine the role of the PDZ domain during Wnt-dependent signalling by Dishevelled, we established stable DVL TKO lines expressing various DVL2–GFP mutants at physiological levels, to assess their rescue activity (henceforth called Rescue_stable_ assay). For comparison, we also tested these mutants after transient transfection of these cells, i.e. under conditions of overexpression (henceforth called Rescue_OE_ assay). Described below is the use of the β-catenin-dependent reporter to assess the rescue activity of DVL2 as, of the five read-outs monitored (Fig. 1), the transcription assay proved to be the most quantitative and sensitive.

**Fig. 3.**
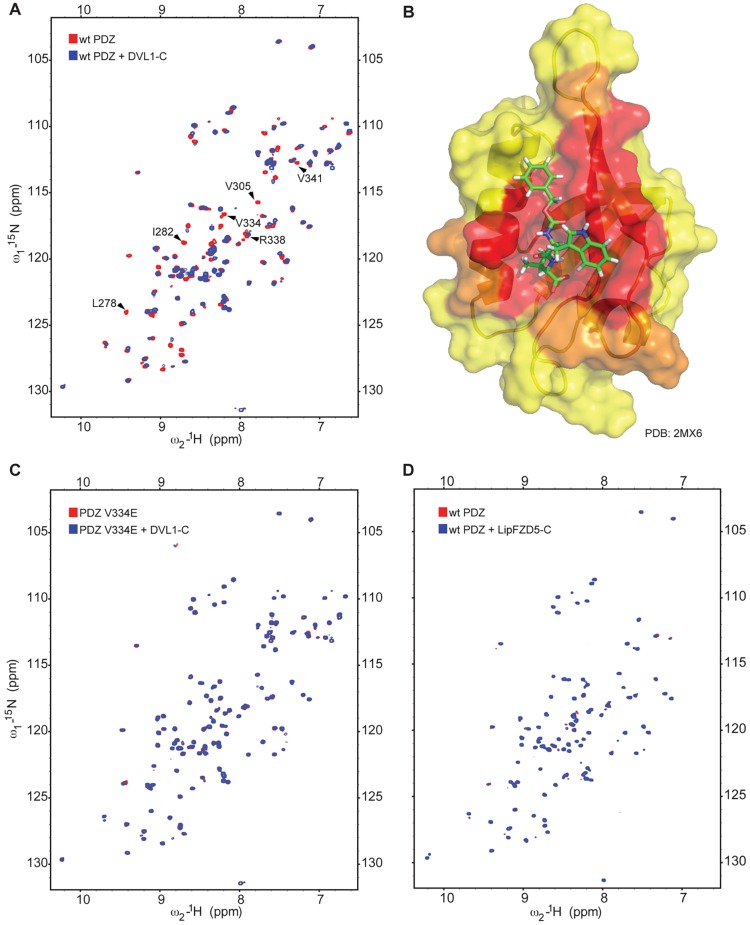
**PDZ cleft mutation blocks DVL-C peptide binding to PDZ.** (A) Overlay for HSQC spectra of 100 µM wt ^15^N-PDZ alone (red) or with 300 µM DVL-C (blue); line broadening and chemical shift perturbation induced by DVL-C peptide affect predominantly PDZ cleft residues (labelled). (B) Heat-map of line broadening [red, >mean+1 standard deviation (1σ) fractional reduction of peak height] and chemical shift perturbation (orange, >mean+1σ |Δδ_1H_|+|Δδ_15N_/5|) projected onto the solution structure (2MX6; Lee et al., 2015) of Dvl1 PDZ (in surface representation) in complex with DVL-C peptide (in stick representation). (C) Spectral overlay as in A, of 100 µM ^15^N-PDZ-V334E alone (red) or with 300 µM DVL-C (blue). (D) Overlay for BEST-TROSY spectra of 100 µM wt ^15^N-PDZ alone (red) or with 300 µM Lip–FZD5 (blue). Spectral overlays in C,D indicate lack of interaction in both cases.

To test whether the PDZ domain is required for DVL rescue activity, we designed two point mutations in its ligand-binding cleft (V334E and D331A R338A, called DARA; see below) that are expected to block ligand access, based on structural information on this cleft and its interaction with cognate physiological ligands (Cheyette et al., 2002; Lee et al., 2015). To confirm that these mutations affect ligand binding, we used a sensitive *in vitro* binding assay based on nuclear magnetic resonance (NMR) spectroscopy, which is capable of detecting low-affinity binding in the high micromolar *K*_d_ range. We purified ^15^N-labelled minimal wt or mutant PDZ domains of DVL2 after bacterial expression, and acquired ^1^H–^15^N correlation spectra, alone or after incubation with a well-validated C-terminal peptide from Dvl1 (DVL-C; Lee et al., 2015). As expected, this peptide induced exchange broadening or shift perturbation of numerous cross peaks in the spectrum of the wt domain (Fig. 3A), as previously observed (Lee et al., 2015). Projection of these affected residues onto the solution structure of the Dvl1 PDZ domain in complex with its DVL peptide ligand (2MX6; Lee et al., 2015) confirmed that these residues are located predominantly in the αB/βB peptide-binding groove (Fig. 3B). In contrast, we only observed weak exchange broadening and shift perturbation of a subset of these cross peaks in the case of the DARA mutant domain upon incubation with excess DVL-C peptide (Fig. S2), confirming that the ligand binding of this mutant is severely compromised, as previously shown (Lee et al., 2015). However, the V334E mutant domain exhibited no exchange broadening or shift perturbation whatsoever upon incubation with peptide (Fig. 3C), which demonstrates that the V334E point mutation completely blocks ligand binding to the DVL2 PDZ domain. In both cases, the spectra show a pattern of peak dispersion similar to that of the wt domain (Fig. 3B; Fig. S2), confirming that the overall structural fold of the PDZ domain is not affected by any of these point mutations. We also tested a Lipoyl (Lip)-tagged peptide spanning the KTxxxW motif from FZD5 (GKTLESWRRFTS) for binding to Lip–PDZ, but failed to see any spectral changes (Fig. 3D). An equally negative result was obtained with an equivalent Lip-tagged peptide from FZD8 (GKTLESWRALCTR; data not shown), or with the peptide from *Xenopus* Fz7 (GKTLQSWRRFYH; Fig. S2) previously tested for interaction with Dvl1 PDZ (Wong et al., 2003). Our failure to detect any interaction of these Frizzled peptides with DVL2 PDZ argues against these cytoplasmic tail sequences of Frizzled being direct ligands for the PDZ domain.

**Fig. 4.**
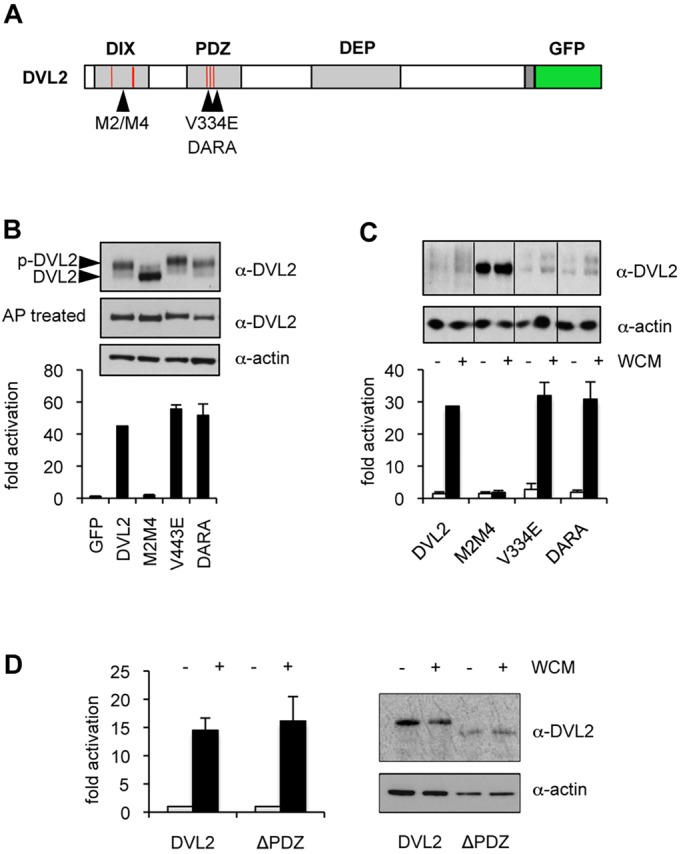
**The PDZ domain is dispensable for Wnt signal transduction to β-catenin.** (A) Cartoon of DVL, with DIX and PDZ mutations indicated. (B,C) Graphs show SuperTOP assays in DVL TKO or control cells transiently (B) or stably (C) transfected with wt or mutant DVL2–GFP, with or without 6 h of incubation in WCM. Above, corresponding western blots. AP, treatment with alkaline phosphatase. (D) Graph shows SuperTOP assays of DVL TKO cells stably transfected with wt or ΔPDZ (Δ244–353) DVL2–GFP as in C. Error bars represent mean±s.e.m., *n*=3 independent experiments. Right panel, corresponding western blots.

Having confirmed that the ligand binding of DVL2 PDZ is blocked by V334E and severely reduced by DARA, we proceeded to test whether these point mutations affect the rescue activity of DVL2. As a benchmark, we included a DIX domain mutant that blocks Dishevelled polymerisation (M2M4; Fig. 4A) (Schwarz-Romond et al., 2007a). As expected, M2M4 was completely inactive in Rescue_OE_ assays, despite its relatively high expression levels; however, V334E and DARA responded to Wnt in the same way as wt DVL2–GFP (Fig. 4B). Essentially the same results were obtained in Rescue_stable_ assays in which M2M4 provided no signalling activity whatsoever, whereas V334E and DARA restored full activity, like wt DVL2–GFP (Fig. 4C). To rule out that these rescue activities reflect residual ligand-binding activity of these cleft mutants, we also tested a deletion mutant of DVL2–GFP that lacks its entire PDZ domain (Δ244–353, ΔPDZ) in our complementation assays. Indeed, the Wnt response of ΔPDZ proved indistinguishable from that of wt DVL2–GFP (Fig. 4D), confirming our results with the PDZ point mutants. These experiments establish that the PDZ domain does not play an essential role in the Wnt signal transduction to β-catenin.

### DEP-dependent DVL2 binding to Frizzled is essential for Wnt signal transduction

**Fig. 5.**
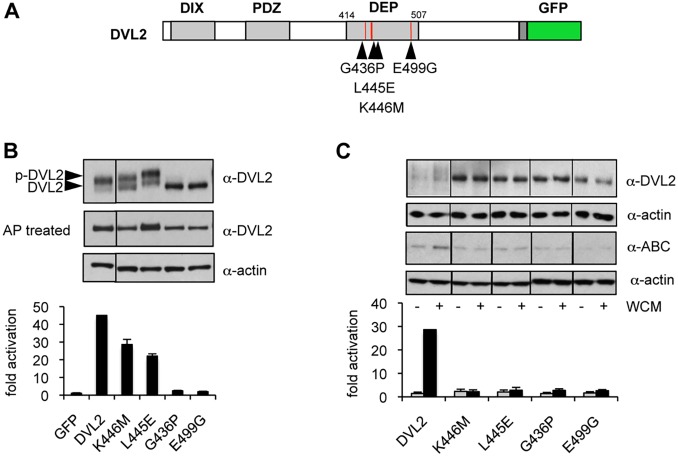
**The DEP domain is essential for Wnt signal transduction to β-catenin.** (A) Cartoon of DVL, with DEP mutations indicated. (B,C) Graphs show SuperTOP assays in DVL TKO or control cells transiently (B) or stably (C) transfected with wt or mutant DVL2–GFP, as in Fig. 3B,C. Error bars represent mean±s.e.m., *n*=3 independent experiments. Above, corresponding western blots.

**Fig. 6.**
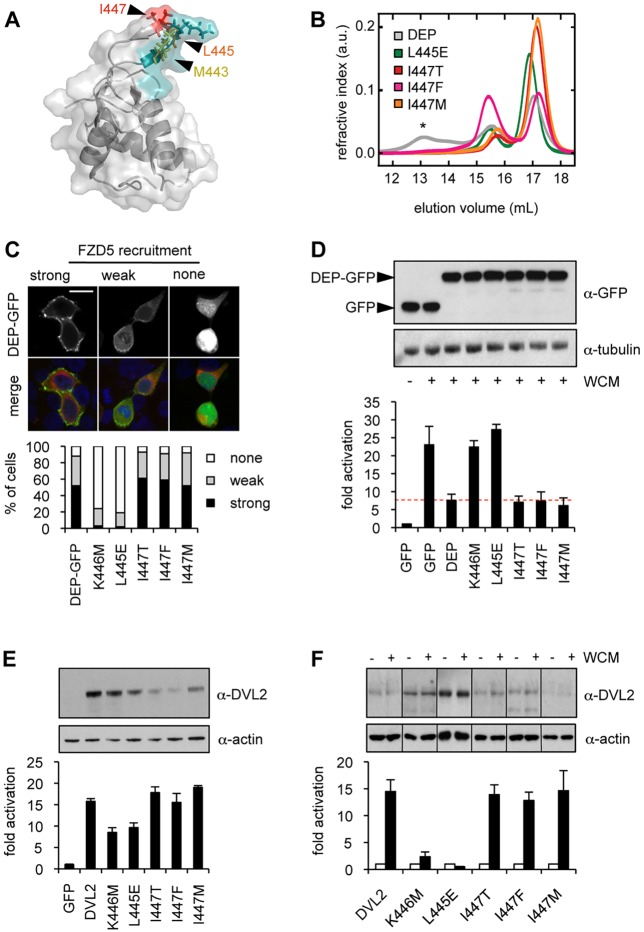
**DEP tetramerisation is not essential for Wnt signal transduction.** (A) Ribbon representation of DEP monomer structure (Protein Data Bank ID number 1FSH), with hydrophobic residues in the tetramerisation interface (in stick) labelled (see also Fig. S4). Turquoise, DEP finger residues required for Frizzled binding (including M443, mutated in *Drosophila dsh^1^*). (B) SEC-MALS of purified wt and mutant DEP domains. Elution profiles of unfractionated Lip–DEP_417-511_ (grey) revealing multiple species of monomers, dimers and tetramers (*). Tetramerisation mutants (colours) elute as monomers and dimers only. (C) Graph shows quantitation of FZD5-dependent recruitment of wt or mutant DEP–GFP to the plasma membrane (100 cells scored in each case). Above, representative images of HEK293T cells showing strong, weak and no recruitment of DEP–GFP. (D) Graph shows SuperTOP assays in transiently transfected HEK293T cells (as in Fig. 1C), showing blocking of endogenous Wnt response by overexpressed wt or mutant DEP–GFP (or GFP as internal control). Above, corresponding western blots. (E,F) Graphs show SuperTOP assays in DVL TKO or control cells transiently (E) or stably (F) transfected with wt or mutant DVL2–GFP, as in Fig. 3B,C; Error bars represent mean±s.e.m., *n*=3 independent experiments. Above, corresponding western blots.

DEP-dependent dimerisation of DVL2 is essential for signalosome assembly, as was revealed by point mutations in the DEP domain that block its dimerisation (including G436P and E499G; Gammons et al., 2016). A different set of DEP mutations block the binding of Dishevelled to Frizzled, but these did not affect its signalling activity in functional assays based on overexpression, as expected given that these assays bypass the need for Wnt stimulation. We thus selected two of our DEP mutants that block recruitment of the minimal DEP domain to FZD5 (L445E, K446M; Gammons et al., 2016) and generated stable lines expressing these mutants, for complementation assays in DVL TKO cells. Both mutants bear single amino acid substitutions in the Frizzled-binding ‘finger’ of the DEP domain (Fig. 5A; see also Fig. 6A); K446M corresponds to the *dsh^1^* mutation in *Drosophila* that causes PCP defects in flies (Axelrod et al., 1998; Perrimon and Mahowald, 1987). We then performed Rescue_OE_ and Rescue_stable_ assays to compare their rescue activities with those of G436P and E499G that block DEP-dependent dimerisation.

As expected, both dimerisation mutants proved to be completely inactive in both rescue assays, regardless of expression levels (Fig. 5B,C). In contrast, L445E and K446M provided substantial rescue activity in Rescue_OE_ assays (Fig. 5B); however, neither mutant exhibited any activity in Rescue_stable_ assays, despite being expressed at substantially higher levels than wt DVL2–GFP – neither mutant was able to induce stabilization of β-catenin, nor β-catenin-dependent transcription, in response to Wnt3a (Fig. 5C). This indicates that, in our physiological complementation assay, the activity of DVL2 to transduce the Wnt signal to β-catenin crucially depends on its DEP-dependent recruitment to Frizzled.

To confirm that the L445E and K446M mutations block the binding of DVL2 to Frizzled, we tested their effects on full-length DVL2–GFP in a recruitment assay based on co-overexpression with SNAP–FZD5 in HEK293T cells (see also Tauriello et al., 2012), alongside the other mutations used in this study. Of all seven mutations tested, L445E and K446M are the only ones that block recruitment of DVL2–GFP to SNAP–FZD5, whereas the other mutations (including V334E and DARA) retain efficient recruitment to membrane-associated SNAP–FZD5, indistinguishably from wt DVL2–GFP (Fig. S3). Thus, the DEP domain is essential for the recruitment of Dishevelled to Frizzled, whereas the PDZ and DIX domain are dispensable.

### DEP tetramerisation is dispensable for Wnt signal transduction

Mutation of L445 not only blocks DEP recruitment to FZD5, but also DEP-dependent tetramerisation, which is a consequence of DEP dimerisation by domain swapping (Gammons et al., 2016); this leucine residue is located at the tip of an exposed loop of the DEP domain (called ‘DEP finger’; turquoise, Fig. 6A), which is required for recruitment to Frizzled. However, this loop can change conformation, by extending to swap the N-terminal α-helix of DEP with that of a second DEP monomer (Fig. S4), thus forming a β-sheet that connects two DEP monomers. Some of the residues in this β-sheet have hydrophobic side-chains (including the hydrophobic triad M443, L445, I447) that mediate association of two dimers into a tetramer. Each of these residues also functions in the monomer to mediate DEP binding to Frizzled (Gammons et al., 2016). This complicates the interpretation of mutations in these residues, e.g. of L445E, which is defective for both Frizzled binding and tetramerisation.

To resolve this, and to determine whether DEP tetramerisation is required for Wnt-dependent signalling in DVL TKO cells, we aimed to substitute residues that are involved selectively in tetramerisation but not in Frizzled binding. The best candidate is I447 whose side chain contributes two key hydrophobic interactions to the DEP tetramerisation interface (owing to its inherent symmetry; Fig. S4), but which occupies a relatively peripheral position within the Frizzled-binding DEP finger (Fig. 6A, red). We thus generated three relatively conservative substitutions of I447, altering it to a residue with a small hydrophilic (I447T) or large hydrophobic side-chain (I447M or I447F), aiming to retain Frizzled binding. Indeed, each of these mutations blocked tetramerisation of purified recombinant DEP domain in size-exclusion chromatography with multi-angle light scattering (SEC-MALS) assays while retaining at least partial dimerisation (Fig. 6B). Conversely, the tetramerisation of K446M is normal (not shown), as expected, given that the positively-charged side chain of K446 remains fully solvent-exposed in the tetramer and thus cannot participate in DEP tetramerisation (Fig. S4).

Next, we introduced these I447 substitutions into DEP–GFP, to test Frizzled binding in two assays based on wt HEK293T cells, as previously described (Pan et al., 2004; Tauriello et al., 2012; Gammons et al., 2016). Indeed, all three mutants were recruited as efficiently as wt DEP–GFP to plasma-membrane-associated SNAP–FZD5 (Fig. 6C), and they also retained full activity in blocking the Wnt-dependent signalling activity of endogenous Dishevelled by interfering with its recruitment to Frizzled (Fig. 6D). By comparison, K446M and L445E were inactive in both assays (Fig. 6C,D), reconfirming that these two mutations block Frizzled binding (see also Fig. S3), as previously shown (Gammons et al., 2016). We conclude that DEP tetramerisation can be blocked selectively without affecting Frizzled binding by all three I447 substitutions tested.

We thus introduced these isoleucine substitutions into DVL2–GFP, for complementation assays in DVL TKO cells. We found that each was fully active in both types of rescue assays, regardless of their expression levels (Fig. 6E,F), in contrast to L445E and K446M whose Wnt-independent signalling activities were somewhat attenuated in Rescue_OE_ assays (Fig. 6E), and reduced to background levels in Rescue_stable_ assays (Fig. 6F), as shown above. This reinforces our conclusion that the DEP-dependent binding of DVL2 to Frizzled is crucial for signal transduction to β-catenin; however, signalling can evidently proceed normally without DEP-dependent tetramerisation.

### Frizzled binding but not dimerisation is required for Frizzled downregulation by Dishevelled

**Fig. 7.**
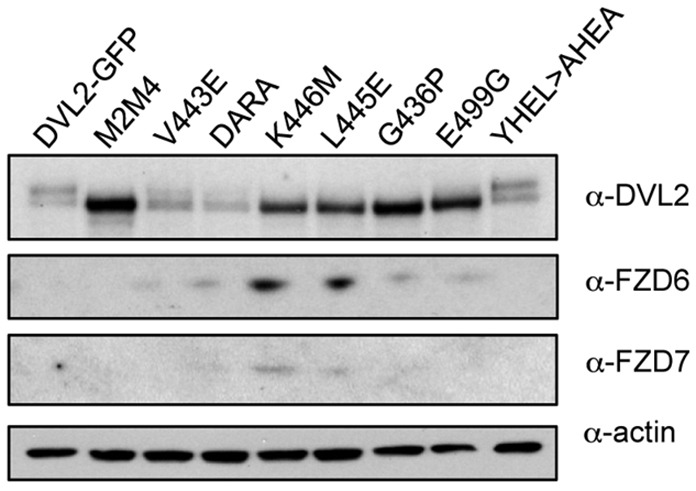
**DEP dimerisation is dispensable for Frizzled downregulation.** Western blots of lysates of DVL TKO cells stably transfected with wt or mutant DVL2–GFP, probed with antibodies as indicated on the left.

In the absence of Wnt signalling, Dishevelled promotes the downregulation of Frizzled and its clearance from the plasma membrane, and this depends on its DEP domain (Jiang et al., 2015). To identify the molecular property of the DEP domain required for this downregulation, we tested our panel of DVL2–GFP mutants for their ability to restore low levels of Frizzled in DVL TKO cells, assessing the levels of endogenous FZD6 and FZD7 in the various stable cell lines. This revealed that the only mutants unable to restore downregulation of Frizzled were K446M and L445E (Fig. 7), i.e. the only two mutants whose recruitment to membrane-associated FZD5 is blocked (Fig. 6C; Fig. S3). By contrast, the dimerisation-defective DEP mutants are both proficient in restoring low levels of FZD6 and FZD7, like the PDZ cleft mutants, the polymerization-deficient DIX mutants, and a mutant whose YHEL motif downstream of the DEP domain was substituted to AHEA (Yu et al., 2007, 2010) to block DVL2 binding to the clathrin adaptor AP2µ (Fig. 7). We conclude that the downregulation of Frizzled by Dishevelled depends purely on the DEP-dependent interaction between the two proteins, but neither on the competence of Dishevelled to signal (i.e. on its DEP-dependent dimerisation and DIX-dependent polymerisation) nor on its ability to bind to the AP2μ clathrin adaptor.

Throughout our study, we noted a striking inverse correlation between the signalling activities of the DVL2–GFP mutants and their expression levels in stable DVL TKO cells lines, whereby the expression levels of all signalling-competent mutants are limited to the low physiological levels seen for wt DVL2–GFP (Fig. 7; see also Fig. 2B). By contrast, the expression levels of the signalling-defective mutants are boosted to high levels (Fig. 7), suggesting that these mutants are subject to positive selective pressure during the establishment of stable cell lines. If so, this implies that these mutants might retain residual function.

## DISCUSSION

We have developed a complementation assay in a human cell line lacking all three DVL paralogs, to test the function of wt and mutant DVL2 stably expressed at near-endogenous level. This DVL TKO line is unable to respond to Wnt stimulation, but Wnt signal transduction to β-catenin is fully restored by a DVL2 transgene whose rescue activity is strictly Wnt-dependent. This assay has enabled us to demonstrate that the DEP domain is essential for Wnt signalling to β-catenin, whereas the PDZ domain is dispensable for this response. This fundamental function of the DEP domain in mediating activation of β-catenin was overlooked in previous functional assays as these relied on Dishevelled overexpression, which bypass the requirement for Wnt-activated receptor signalling. Its essential role in mediating Dishevelled recruitment to Frizzled was uncovered by our assay for the first time.

Undoubtedly, the binding of Dishevelled to Frizzled receptor is key to its function in Wnt signal transduction. This was initially thought to be mediated by the PDZ domain, based on an *in vitro* interaction of purified domain with a peptide spanning the functionally relevant KTxxxW motif derived from the cytoplasmic tail of Frizzled (Wong et al., 2003). However, in the context of full-length Frizzled, these peptides are likely to form an α-helix adjacent to the seventh transmembrane domain and along the lipid bilayer, as in most known GPCRs structures (Venkatakrishnan et al., 2013) including the close Frizzled relative Smoothened (Wang et al., 2013). The notion of this peptide adopting a helical structure is further supported by experimental evidence on micelle-binding peptides from FZD1 (Gayen et al., 2013). If so, this is incompatible with recognition by PDZ domains; these domains require their ligands to adopt a β-strand-like conformation to form a β-sheet with a β-strand in their cognate binding clefts (Ye and Zhang, 2013) in a so-called β-augmentation, also seen in DVL-PDZ–ligand binding (Cheyette et al., 2002; Lee et al., 2015; Ye and Zhang, 2013; Zhang et al., 2009). Furthermore, although the DVL PDZ domain has an unusually versatile binding cleft, accommodating a bewildering range of sequence-unrelated C-terminal and internal peptides (Zhang et al., 2009), the only well-documented cases of PDZ structures in complex with physiologically relevant ligands represent C-terminal peptides of the negative Wnt regulator Dapper (Cheyette et al., 2002) and of Dvl1 itself (Lee et al., 2015). Indeed, we were unable to detect any binding between KTxxxW-spanning peptides from three different Frizzled paralogs (including FZD5) and the DVL2 PDZ domain in our NMR binding assay (Fig. 3D; Fig. S2). Thus, the cumulative structural and biophysical evidence argues strongly against the notion that the Dishevelled PDZ domain binds directly to Frizzled, consistent with our findings that this domain is also dispensable for Wnt-dependent signal transduction to β-catenin. In other words, the physiological role of the Dishevelled PDZ domain is yet to be demonstrated; its main function might be to downregulate signalling through binding to negative Wnt regulators such as Dapper and Naked (Cheyette et al., 2002) or to the C-terminus of Dishevelled itself (Lee et al., 2015), or to transduce non-canonical Wnt signals, as indicated by functional studies in flies (Axelrod et al., 1998).

Instead, it seems far more plausible that Frizzled binding is achieved by the DEP domain. Evidence for this first emerged from biophysical studies revealing a direct interaction between purified DEP domain and peptides derived from the cytoplasmic face of Frizzled, including its C-terminal tail (Tauriello et al., 2012). Furthermore, the DEP domain is required for Dishevelled translocation to membrane-associated Frizzled, and also blocks Wnt signal transduction by endogenous Dishevelled upon overexpression (Cong et al., 2004; Pan et al., 2004; Tauriello et al., 2012; Strutt et al., 2012; Gammons et al., 2016). Indeed, the single-most important result of our current study is the demonstration that the DEP-dependent recruitment of Dishevelled to Frizzled is essential for Wnt signal transduction to β-catenin in human cells, based on the K446M mutant whose sole defect is in Frizzled binding. In previous overexpression assays, this mutant retained efficient signalling activity (Boutros et al., 1998; Li et al., 1999; Moriguchi et al., 1999; Tauriello et al., 2012; Gammons et al., 2016); however, it proved to be completely inactive in our physiological complementation assay. This result provides powerful support for the notion that the DEP domain harbours a fundamental function in Wnt signal transduction – the binding of Dishevelled to Frizzled.

A distinct yet equally important function residing in the DEP domain is its ability to dimerise, uncovered by a separate set of point mutations that block dimerisation (Gammons et al., 2016). DEP-dependent dimerisation triggers signalosome assembly, probably by cross-linking DIX-dependent Dishevelled polymers, and is essential for both Wnt-dependent and Wnt-independent signalling by Dishevelled. Therefore, unlike the DEP-dependent binding to Frizzled, this dimerisation function is detectable in overexpression assays, explaining previous evidence for the fundamental importance of the DEP domain in Wnt signalling (e.g. Axelrod et al., 1998; Li et al., 1999; Moriguchi et al., 1999; Rothbacher et al., 2000; Penton et al., 2002; Wang et al., 2006; Strutt et al., 2012). DEP dimerisation is not required for Frizzled binding, and structural analysis revealed that these two molecular interactions are mutually exclusive and depend on distinct conformations of the DEP domain. Indeed, the residues required for Frizzled interaction are masked in the DEP dimer, and engage in DEP tetramerisation, which is an inevitable consequence of DEP dimerisation (Gammons et al., 2016). In our current study, we have succeeded in generating mutants that block tetramerisation without affecting Frizzled binding, but these provided full rescue activity in our complementation assay. Although the tetramerisation by the DEP domain could ensure the unidirectionality of signalling by blocking the re-binding of Dishevelled to Frizzed after catalysing signalosome formation, this function is clearly non-essential in our assay and might only be detectable in more sensitive functional tests.

Our complementation assay also uncovered a Wnt-independent function of the DEP domain in downregulating the steady-state levels of Frizzled, probably through ubiquitin E3 ligases RNF43 and/or ZNRF3 (Jiang et al., 2015), which is independent of its ability to dimerise. This function also seems to be independent of a direct interaction between Dishevelled and the AP2µ clathrin adaptor (Fig. 7), arguing against a direct role of Dishevelled in coupling ubiquitylated Frizzled with clathrin-dependent endocytosis in the absence of Wnt. Indeed, its DEP-dependent binding to Frizzled seems to suffice for this function, suggesting that the role of Dishevelled in this process is that of an adaptor between Frizzled and RNF43 and/or ZNRF3.

One of our most striking observations was that the expression levels of wt and signalling-competent DVL2 mutants were kept low, to a level similar to that of endogenous DVL, indicating that these levels are subject to negative selective pressure. This can be explained by our finding that cell cycle progression is blocked if DVL2 is left to accumulate to high levels, although we have not investigated the molecular basis for this phenomenon. Conversely, the expression levels of our inactive DVL2 mutants were universally high, possibly as a result of positive selective pressure; clearly, absence of Dishevelled slows down the rate of cell proliferation (Fig. 1D), and the process of isolating stably transfected cell lines might favour cells whose proliferation rate is slightly enhanced if their rescue transgene retained residual function. It is possible that our point mutations do not fully inactivate DVL2, which could also explain why *dsh^1^* (corresponding to K446M) sustains development to viable flies without a detectable defect in signalling to *Drosophila* β-catenin (Axelrod et al., 1998; Perrimon and Mahowald, 1987). Likewise, CRISPR–Cas9-engineered dimerisation mutations of the endogenous fly *dsh* gene (equivalent to G436P and E499G) are viable (J. Mieszczanek, MRC Laboratory of Molecular Biology, Cambridge, UK and M.G., unpublished observations). It thus seems that normal development in flies requires only a fraction of the wt function of its Dishevelled DEP domain.

Our complementation assay also allowed us to establish the epistatic relationship between Dishevelled and LRP6. Initially, epistasis analysis in *Drosophila* suggested that Dishevelled functions below LRP6 (Wehrli et al., 2000), but this was subsequently revised to the converse (Metcalfe et al., 2010), reconciling the fly data with results from vertebrate systems showing that the Wnt-dependent phosphorylation of LRP6 depends on Dishevelled (Bilic et al., 2007). Indeed, the signalling activity of DVL2 was found to be much reduced in LRP6-depleted cells (Metcalfe et al., 2010). We now use null-mutant human cells to establish that the overexpressed LRP6 is equally active whether or not Dishevelled is present, whereas overexpressed DVL2 barely signals in the complete absence of LRP6. This identifies LRP6 as an essential signalling effector of Dishevelled, transducing the Wnt signal from Dishevelled to β-catenin.

In summary, our complementation assay provides a test system for the Wnt-dependent signalling activity of Dishevelled, which enabled us for the first time to demonstrate the essential role of the DEP domain in mediating Frizzled binding by Dishevelled for its activity in Wnt signal transduction to β-catenin. It also provides an assay system for other functions of Dishevelled, such as the downregulation of Frizzled and the progression of the cell cycle, which we have not further pursued. Indeed, it might be adaptable to the study of non-canonical functions of Dishevelled although this might require the supply of additional co-factors, the use of different cell types or even organoids. It might thus pave the way for the development of similar assay systems that combine loss-of-function with stable re-expression of rescue transgenes at physiological levels, yielding definitive functional insights.

## MATERIALS AND METHODS

### Plasmids and antibodies

The following plasmids were used: human DVL2–GFP (pEGFP-N1); SNAP–FZD5 (Koo et al., 2012); LRP6–GFP (Metcalfe et al., 2010); PX330-spCas9 (Addgene, #43330) for DVL TKO; PX458-spCas9-GFP (Addgene, #48138) for LRP6 KO. DVL2–GFP was subcloned into pBabe PURO (Addgene) to generate stable cell lines. DVL2 mutants were generated by standard procedures and verified by sequencing. The following antibodies were used: anti-tubulin (1:5000; T4026, Sigma), anti-GFP (1:5000; G1544, Sigma), anti-actin (1:5000; ab8227, Abcam), anti-FZD3 (1:2500; ab75233, Abcam), anti-FZD6 (1:5000; ab128916, Abcam), anti-FZD7 (1:1000; ab64636, Abcam), anti-FZD8 (1:3000; ab40012, Abcam), anti-SNAP (1:5000; P9310S, NEB), anti-DVL1 (1:1000; sc8025, Santa Cruz), anti-DVL3 (1:1000; sc26506, Santa Cruz), anti-DVL2 (1:5000; 3216, Cell Signaling), anti-ABC (1:5000; 8814, Cell Signaling), anti-p-LRP6 S1490 (1:2500; 2568, Cell Signaling).

### Isolation of stable cell lines

To generate DVL TKO cells, HEK293T cells (Chiu et al., 2016; not authenticated) were transfected with plasmids encoding Cas9 and guide RNAs (Ran et al., 2013) targeting genomic loci of *DVL* (*DVL1/2*, 5′-CTACATTGGCTCCATCATGA; *DVL3*, 5′-ACCATGCTTCAATGGCCGGG) or *LRP6* (*LRP6*, 5′-CGATTGGTTGATGCTACAAA); clones were screened by immunoblotting, and deletions were confirmed by genomic DNA sequencing. To generate stable DVL2-expressing cell lines, DVL TKO cells were transfected with 200 ng DVL2–GFP pBabePURO:PEI (1:3), and selected with 2 µg/ml puromycin.

### Cell-based assays

HEK293T cells were cultured and transfected as described (Mund et al., 2015), and single confocal images were acquired at identical settings with a Zeiss confocal microscope. For SuperTOP assays (Veeman et al., 2003), HEK293T cells were lysed 16–24 h after transfection, and analysed with the Dual-Glo Luciferase Reporter Assay (Promega) according to the manufacturer's protocol. Experiments were repeated three times, and values were normalised to Renilla luciferase (shown as mean±s.e.m. relative to vector controls). Wnt3a-conditioned media (WCM) were generated from L-cells (ATCC CRL-2647) according to manufacturer's specification. Alkaline phosphatase treatments (Roche), used in some experiments to dephosphorylate DVL, were done for 30 min at 37°C. For proliferation assays, 3×10^4^ cells were seeded, and cells were counted every 24 h (in triplicate, three independent experiments); results were analysed by two-way ANOVA with repeated measures, and are shown as mean±s.e.m.

### Protein purification and biophysics

DVL2 DEP_417-511_, DVL2 PDZ_265-361_, FZD5 (GKTLESWRRFTS) and FZD8 peptide sequences (GKTLESWRALCTR) were tagged N-terminally with 6×His and Lip followed by a linker (ENLYFQS) encoding a cleavage site for tobacco etch virus (TEV) protease. Lip–Fz7 (encoding GKTLQSWRRFYH; Wong et al., 2003) was generated similarly, except that the TEV linker sequence was flanked by serine–glycine on both sides. Proteins were expressed in *E. coli* BL21-CodonPlus(DE3)-RIL cells (Stratagene) at 37°C, grown overnight at 24°C, and induced with isopropyl β-D-1-thiogalactopyranoside at OD 0.8. Lip–DEP, Lip–PDZ and Lip–FZDs were purified on NiNTA beads (Qiagen) essentially as described (Fiedler et al., 2011). SEC-MALS was performed in phosphate-buffered saline, using a GE Superdex S-200 10/300 analytical column, and analysed as described (Madrzak et al., 2015).

### NMR spectroscopy

Minimal wt and mutant ^15^N-labelled PDZ domains were purified essentially as described (Fiedler et al., 2011), and used for NMR spectroscopy after cleaving off the Lip tag (with TEV protease overnight at 4°C). NMR spectra were acquired on a Bruker Avance-III spectrometer operating at 600 MHz ^1^H frequency, and equipped with a cryogenic inverse 5 mm probe at a sample temperature of 298 K. All samples were prepared in aqueous phosphate buffer at physiological ionic strength (pH 6.7). Backbone resonance frequencies were obtained for 500 µM ^13^C,^15^N-labelled protein, using unmodified Bruker pulse programmes for HNCACB, CBCA(CO)NH, HN(CA)CO and HNCO. BEST-TROSY (Favier and Brutscher, 2011) or fast-HSQC ^1^H-^15^N correlation spectra were acquired for purified ^15^N-PDZ with 128 complex and 1024 points (1.1 and 0.7 Hz/point in the processed data) in *t*_1_ and *t*_2_, respectively, and 8 transients per *t*_1_ point. In each case, 100 µM PDZ protein was incubated with 3× molar excess of DVL-C (SEFFVDVM; Lee et al., 2015; kindly provided by Jie Zheng, Department of Ophthalmology, University of California, Los Angeles, USA) or Lip–FZD peptides (as indicated in Fig. 3 and Fig. S2).
